# *Chlamydia psittaci* genotype B in a pigeon (*Columba livia*) inhabiting a public place in San José, Costa Rica

**Published:** 2013-12-02

**Authors:** G. Dolz, Á. Solórzano-Morales, L. Angelova, C. Tien, L. Fonseca, M.C. Bonilla

**Affiliations:** *Laboratorio de Entomología y Medicina Poblacional, Escuela de Medicina Veterinaria, Universidad Nacional, P.O. Box 86-3000, Heredia, Costa Rica*

**Keywords:** *Chlamydia psittaci*, Costa Rica, Genotype B, Pigeons, Zoonosis

## Abstract

Human chlamydiosis is a zoonotic disease of avian origin caused by *Chlamydia psittaci*. The highest infection rates have been detected in parrots (*Psittacidae*) and pigeons (*Columbiformes*), the latter most frequently carry the genotypes B and E. These genotypes have been shown to also infect humans. Because pigeons (*Columba livia*) cohabit with humans in urban areas, *C. psittaci* present in the dust from dry feces of infected pigeons may be transmitted by inhalation and represent a significant public health problem. Between 2012 and 2013 a total of 120 fecal samples were collected from pigeons at four public places (Plaza de la Cultura, Parque Morazán, Parque Central de Guadalupe, Plaza de las Garantías Sociales) in San José, Costa Rica. A nested polymerase chain reaction (PCR) was used to amplify a region of the outer membrane protein A gene of *C. psittaci*. Only one sample was positive in PCR and the positive sample was further subjected to sequencing and genotyping. Sequencing identified this sample as *C. psittaci* genotype B. This study is the first report to show the presence of this organism in pigeons of Costa Rica, and shows that the infected pigeons may represent a significant risk for humans who visit public places that are inhabited by pigeons.

## Introduction

Avian chlamydiosis is caused by *Chlamydia psittaci* (*C. psittaci*), a gram-negative and obligate intracellular bacterium, with nine (A to F, E/B, M56, and WC) known genotypes (Van Lent *et al.*, 2012). *C. psittaci* has been identified in 465 different bird species (Kaleta and Taday, 2003), but the highest rate of infection was found in parrots (*Psittacidae*) and pigeons (*Columbiformes*) (de Freitas *et al.*, 2002; Dovc *et al.*, 2005; Dovc *et al.*, 2007).

*C. psittaci* is shed regularly or intermittently in feces, lacrimal fluids, nasal discharges, oropharyngeal mucus and crop milk of the infected birds. Extreme environmental changes can trigger the onset of clinical disease in the infected birds; however prolonged subacute clinical forms of the disease are most common (Gerlach, 1986). Avian chlamydiosis presents in the form of nonspecific clinical signs to severe systemic disease, especially in young animals (Andersen and Vanrompay, 2003).

All genotypes of *C. psittaci* can be transmitted to humans and cause psittacosis or parrot fever (Andersen and Vanrompay, 2003); this transmission can occur either through inhalation, ingestion or via direct contact with the infected birds (Longbottom and Coulter, 2003). In humans, the disease can vary from nonspecific flu-like symptoms to severe pneumonia. Also, cases of endocarditis and encephalitis have been reported (Crosse, 1990).

*C. psittaci* is the most common pathogen found in domestic pigeons and mainly belong to the genotypes B and E (Vanrompay *et al.*, 1993; Andersen and Vanrompay, 2000; Magnino *et al.*, 2009). Because pigeons spread chlamydial infection and usually fly distances of about 5 km, these birds represent a major risk to public health as they cohabit with humans in urban and rural areas, in public places, parks and even gardens. It is believed that pigeons have been underestimated for a long time as an important source of human chlamydial infections (Heddema *et al.*, 2006a; Vázquez *et al.*, 2010).

A study carried out in Costa Rica in 2001 found antibodies against *C. psittaci* in 12.4% of 129 captive macaws (*Ara macao* and *Ara ambigua*) (Herrera *et al.*, 2001). Recently *C. psittaci* was detected in four (3.4%) out of 117 captive psittacine birds, which cohabited in human households and in two of those cases the organism was found to be of genotype A (Sheleby-Elias, 2010).

The objective of the present study was to determine the presence of *C. psittaci* in feces of pigeons of urban public places and parks that are frequently visited by children, elderly and tourists.

## Materials and Methods

### Size, type of sample and sampling method

Between October 2012 and May 2013 a total of 120 pigeons were tested in four different public parks (Plaza de la Cultura, Plaza de las Garantías Sociales, Parque Morazán and Parque Central Guadalupe) in San José, by cookie crumbs.

Feces were collected from the floor with sterile swabs from defecating birds, and transferred into tubes containing 0.5 ml Minimal Essential Medium. The swabs were stored at 4°C for 24 hours until further processing.

### Polymerase chain reaction (PCR)

For DNA extraction DNeasy® Blood & Tissue Kit of QIAGEN (Venlo, Netherlands) was used and the extraction was performed according to the manufacturer’s instructions. For the detection of *C. psittaci*, a nested PCR described by Sachse and Hotzel (2003) was used, which partially amplifies the gene outer membrane protein A (*ompA*) to identify the genus *Chlamydia*. The primers used were: 191CHOMP (5’-GCIYTITGGGARTGYGGITGYGCI AC-3’) and CHOMP371 (5’-TTAGAAICKGAATTGIGCRTTIAYGTGIGCIGC-3’). All amplification products were subjected to a second PCR to identify *C. psittaci*, using the primers CHOMP 336s (5’-CCRCAAGMTTTTCTRGAYTTCAWYTTGTTRAT-3’) and 218PSITT (5’-GTAATTTCIAGCCCAGCACAATTYGTG-3’). Samples with bands of 389-404 bp were considered positive for *C. psittaci*. DNA of *C. psittaci* was donated by the Clinic of Birds, Reptiles, Amphibians and Fish, Justus Liebig University, Giessen, Germany and was used as a positive control; nuclease free water (molecular biology grade, Thermo Scientific, Waltham, USA) was used as a negative control.

### Sequencing, genotyping and construction of the phylogenetic tree

One positive sample identified during nested PCR was genotyped through analysis of *ompA* sequences (Heddema *et al.*, 2006b).

The primers used were CPsittGenoFor (5’-GCTACGGGTTCCGCTCT-3’) and CPsittGenoRev (5’-TTTGTTGATYTGAATCGAAGC-3’), which amplify conserved regions of the *ompA* gene covering four variable domains. The size of the amplified fragment was 1041bp. PCR product was purified using the QIAquick® kit (QIAGEN, Venlo, Netherlands), according to the manufacturer’s instructions.

The DNA sample was sent to Macrogen (Seoul, Korea) for sequencing. Partial sequence was aligned with BioEdit Sequence Aligment Editor® (Hall, 1999) and compared using the BLASTn algorithm with the database of National Center for Biotechnology Information. Then the sequences were imported into MEGA 5 (Tamura *et al.*, 2011), using the Jukes & Cantor algorithm (Jukes and Cantor, 1969) and the UPGMA method (Sneath and Sokal, 1973), for the design of the phylogenetic tree. A total of 10000 replicates were calculated (Felsenstein, 1985). The analysis included the reference sequences of the nine *C. psittaci* genotypes available in the database of GenBank, A (accession number AY762608), B (AF269265), C (L25436), D (AF269266), E (X12647), F (AF269259), E/B (AY762613), M56 (AF269268) and WC (AF269269) (Sachse *et al.*, 2008), the phylogenetic tree was rooted with the *C. caviae* strain (GPIC, GenBank AF269282) (Zhang *et al.*, 1989).

## Results

**Fig. 1:**
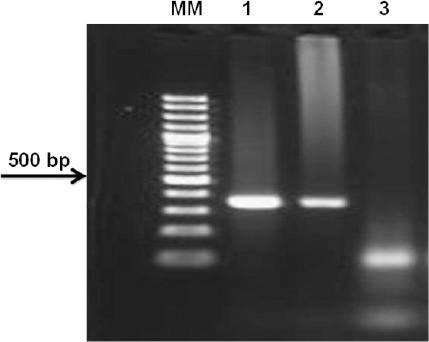
Gel electrophoresis of the amplified PCR products of *omp*A gene of *C. psittaci* (MM: molecular marker; 1: positive control; 2: pigeon from Costa Rica; 3: negative control).

Only one out of 120 samples yielded positive results during the first PCR (576-597pb), and the specific band (389-404pb) was subsequently amplified using the nested PCR (Fig. 1).

**Fig. 2:**
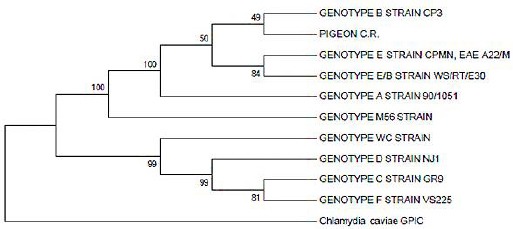
Phylogenetic tree of gene *omp*A sequence of one pigeon sample (PIGEON C.R.) of Costa Rica that tested positive for *C. psittaci*.

The sample that tested positive for *C. psittaci* was collected from a pigeon from the public park Plaza de la Cultura. Sequencing confirmed the results, and identified this sample as *C. psittaci* genotype B (Fig. 2).

**Fig. 3:**
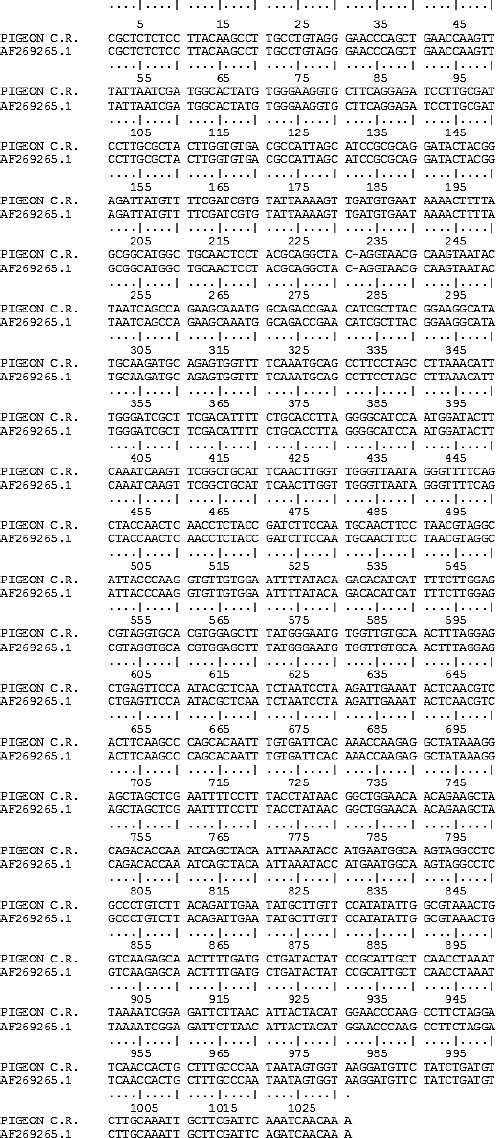
Alignment of the nucleotide sequences of the *omp*A gene sequence of *C. psittaci* obtained from a pigeon in Costa Rica (PIGEON C.R.) with that of *C. psittaci* genotype B (AF269265.1) isolated in 1958 from air sacs of pigeons in California, USA. The nucleotide difference between the sequences is underlined at the position.

The sequence analysis of the product of PCR using BLASTn, found 99.9% (1029/1030) nucleotide identity with sequences of *C. psittaci* genotype B deposited in GenBank (CP003797.1, AY762609.1, AF269265.1). Comparison of the partial sequence of the fecal pigeon sample of Costa Rica with a *C. psittaci* sequence isolated in 1958 from air sacs of pigeons in California, USA by Bush and Everett (2001) is shown in Figure 3.

## Discussion

This is the first study in Costa Rica that reported the presence of *C. psittaci* genotype B in a pigeon of a public park in Costa Rica, and is consistent with the international literature which mentions that this genotype is found more frequently in domestic and feral pigeons (Vanrompay *et al.*, 1993; Andersen and Vanrompay, 2003; Magnino *et al.*, 2009).

Only one bird was found to be positive for the pathogen (3.3%), in contrast to studies carried out in Madrid (61/116, 52.6%), Zagreb (30/232, 12.9%) and Amsterdam (26/331, 7.8%) (Pruckner-Radovcic *et al.*, 2005; Heddema *et al.*, 2006a; Vázquez *et al.*, 2010), but our results are in accordance with studies carried out in Ghent (1/61, 1.6%) (Dickx *et al.*, 2010). The reasons could be due to several factors such as type of the study, sample size, or the sampling method.

In addition, false negative results could not be ruled out, due to polymerase inhibitors in the samples or because of intermittent shedding of *C. psittaci* from cloacal samples that could have resulted in an underestimation of the agent (Pruckner-Radovcic *et al.*, 2005).

Nevertheless, it may also indicate a low infection rate of *C. psittaci* in pigeons of Costa Rica, which would be consistent with studies carried out by Sheleby-Elias (2010), who detected a low infection rate (3.4%) of *C. psittaci* in captive psittacine birds inhabiting the households. The genotype present in captive parrots was determined as genotype A.

The finding of this zoonotic agent in the feces of a pigeon shows that *C. psittaci* is found in places that are regularly visited by human population, especially on weekends by families with young children, who feed the pigeons. This poses a significant risk of transmission of the agent by inhalation or direct contact with the infected birds (Longbottom and Coulter, 2003).

The low infection rate determined in the present study is beneficial to the public health (Pruckner-Radovcic *et al.*, 2005), nevertheless it is recommended that more comprehensive and representative studies are required to determine the prevalence of *C. psittaci* in urban pigeons, establish the genotypes circulating in these birds, because certain genotypes (A and D) can cause serious infections in people, while others, such as genotype B, usually cause mild respiratory symptoms (Dickx *et al.*, 2010).

Finally, we recommend implementation of control programs for decreasing the prevalence of this disease in pigeons, coupled with environmental educational programs, to prevent a possible spread of *C. psittaci* to people visiting public places of Costa Rica.
